# Commentary: Systemic, Local, and Imaging Biomarkers of Brain Injury: More Needed, and Better Use of Those Already Established?

**DOI:** 10.3389/fneur.2016.00034

**Published:** 2016-03-21

**Authors:** Marcelo de Lima Oliveira, Edson Bor-Seng-Shu, Renata Faria Simm, Tatiana Vilas Boas, Paulo Henrique Pires Aguiar

**Affiliations:** ^1^Neurology Department, Hospital Santa Paula, São Paulo, Brazil; ^2^Surgical Department, Universidade Federal do Rio Grande do Sul, São Paulo, Brazil

**Keywords:** cerebral metabolic crises, traumatic brain injury, mitochondrial dysfunction, glucose metabolism, subarachnoid hemorrhage

Cerebral metabolism plays an important role in maintaining cell functions. The brain requires glucose as the main substrate for aerobic glycolysis to sustain the high cerebral metabolic demands. Glucose transport through the blood–brain barrier is mediated by GLUT1 glucose transporter protein. Higher cerebral metabolic needs may increase GLUT1 expression on microvessels, thus enhancing glucose uptake (1). Therefore, glucose is the only substrate that is transported across the BBB at a sufficient rate. Cerebral ischemia is classically defined as a cellular condition in which there is insufficient glucose and oxygen for energy production (2, 3).

Some authors have described cerebral metabolic crisis (MC) that is related to alterations in the use of glucose for energy synthesis, traumatic brain injury (TBI), and subarachnoid hemorrhage (SAH) (4–7). What process does the term “cerebral MC” actually describe? To understand this issue, it is important to identify the two major pathways that produce neuronal energy: (1) an aerobic path in which 1 glucose molecule results in 38 ATP molecules and H_2_O and (2) an anaerobic path in which 1 glucose molecule results in 2 ATP molecules and 2 molecules of lactate. Both of these pathways use glycolysis to convert glucose to pyruvate, which is the main substrate needed to provide energy (8). Thus, energy biomarkers, such as glucose, lactate, and pyruvate, provide relevant information describing cerebral metabolism. During cerebral tissue hypoxia, energy is mainly produced anaerobically. The restoration of cerebral blood flow and oxygenation within a proper time frame leads to the normalization of aerobic cell respiration if the mitochondrial function is still preserved. However, during mitochondrial dysfunction in which neuronal cells are unable to use the available tissue oxygen, the anaerobic (redox) pathway is also used; mitochondrial dysfunction can occur due to or during decreased blood flow and may also occur in the normal blood flow/hyperemia state (2, 6, 9). Both ischemia and mitochondrial dysfunction states produce lactate with high pyruvate consumption, resulting in an increased lactate/pyruvate ratio (L/P) type 1 (10). However, cerebral MC can occur in the absence of high lactate levels and are classified as L/P type 2 characterized by decreased pyruvate synthesis. MCs can occur during a variety of pathological cellular states, such as TBI, SAH, stroke, and meningitis, and may be associated with high or normal levels of oxygen in the cerebral tissue.

Carpenter et al. (3) determined that an increased L/P ratio is a consequence of the redox state. However, there are other cerebral MCs. It is important to reinforce the following concepts regarding MCs: (1) mitochondrial dysfunction is a MC associated with an increased L/P type 1 and redox state, (2) other MCs may be linked to a shift from glucose metabolism to other functions in neuronal cells and are associated with an increased L/P type 2, (3) glucose is not the only substrate used to produce energy in neuronal cells, and (4) different cerebral MCs may occur simultaneously.

Some cerebral MCs are characterized by decreased glucose metabolism. Thus, other substances may be used to produce cerebral cellular energy. Lactate can be oxidized in the brain and may be an energy source. Furthermore, peripheral lactate reaches the liver, where it stimulates glucose production *via* gluconeogenesis and provides glucose to the brain to restore glucose metabolism (11). Moreover, cerebral uptake of lactate increases during intense exercise without high levels of CSF lactate. Thus, lactate is oxidized to be used as fuel, sparing glucose, and is eliminated by the brain, similar to the liver (12). Additionally, in reversible ischemia, LPR normalizes within 60–90 min of CBF restoration; this finding reinforces that accumulated lactate can be aerobically used (8). Another study suggested that ketones are oxidizable substances that may be used as substrates to produce energy and can lead to improved energy status (4, 13, 14). Furthermore, a ketogenic diet reduces the lactate levels and improves the ATP levels after TBI in juvenile rats (13). Therapy with branched chain amino acids after percussion TBI improved synapse efficiency and cognitive performance in adult mice possibly by normalizing brain levels of this amino acid, which has a role in glutamate metabolism (13). Conversely, glucose metabolism may increase during a TBI. In the presence of oxidative stress, glucose metabolism is diverted to the pentose phosphate pathway (PPP). This pathway produces a reduction equivalent in the form of NADPH, which plays a cellular protective role by removing free radicals through its action over glutathione. Ketones may play the same PPP role of increasing antioxidants and scavenging free radicals, which mediate mitochondrial function (15). Glutathione peroxidase is a key antioxidant enzyme that is elevated at 3 h and peaks 7 days post-traumatic brain injury. The PPP also produces ribose, which is important for DNA repair and replication and for mRNA and protein synthesis. The PPP is responsible for approximately 2–5% of glucose utilization during normal metabolism but can reach levels exceeding 8–12% after TBI (2, 16).

During these cited conditions, pyruvate synthesis is reduced, and the L/P type 2 increases (10). These findings suggest that cerebral MCs are not restricted to the condition of high lactate synthesis that occurs during ischemia and mitochondrial dysfunction. Importantly, free radicals, which are associated with ischemia, can cause mitochondrial failure and consequently reduce oxidative metabolism; furthermore, glucose may be used to remove free radicals. Thus, mitochondrial dysfunction and other MCs may coexist.

We emphasize that MCs consist of a broad spectrum of glucose and other energy synthesis disturbances. The diagnoses of different MCs may increase treatment options for “cell symptoms” and could improve patient prognosis.

## Author Contributions

MLO: author and writer, EBSS: general article reviewer, RFS: drafting the work, TVB: reviewer about general metabolic crises treatment, and PHPA: final approval for article publication.

## Conflict of Interest Statement

The authors declare that the research was conducted in the absence of any commercial or financial relationships that could be construed as a potential conflict of interest.
